# Mothers’ dietary diversity and associated factors in megacity Dhaka, Bangladesh

**DOI:** 10.1016/j.heliyon.2023.e19117

**Published:** 2023-08-12

**Authors:** Sadika Haque, Md Salman, Md Sadique Rahman, Abu Torab M.A. Rahim, Md Nazmul Hoque

**Affiliations:** a Department of Agricultural Economics, Bangladesh Agricultural University, Mymensingh, Bangladesh; b Department of Management and Finance, Sher-e-Bangla Agricultural University, Dhaka, Bangladesh; c Institute of Nutrition and Food Science, University of Dhaka, Dhaka, Bangladesh; d Students' Affairs Division, Bangladesh Agricultural University, Mymensingh, Bangladesh

**Keywords:** Dietary diversity, Nutrition, Regression analysis, Urban areas, Women

## Abstract

Mothers in developing countries are nutritionally vulnerable due to an undiversified diet. Dietary diversity and healthy dietary patterns of mothers are necessary for the health and nutrition of both the mother and the child. Keeping these in mind, the study was designed to investigate the determinants of mothers' dietary diversity in the capital city (Dhaka) of Bangladesh. A total 613 mothers who had at least one child aged 6–59 months were surveyed in 2020. Dietary diversity (DD) was measured by 24 h recall period following the established guidelines. To explore the determinants of dietary diversity, a log linear regression model was employed. The findings revealed that the overall DD of mothers was low, with less than 15% of respondents consuming more than 5 of the 9 food groups. The study found that if a mother receives one more year of formal education, her DD, on average, would increase by 0.70%. Receiving antenatal care (ANC) for four or more times during pregnancy increases DD by 5.13% compared to mothers who receive ANC less than four times. The findings also showed that mothers with access to assets have 10.18% higher DD than mothers without access to assets. On the other hand, mothers' employment status was negatively associated with DD. Redistributing the household workload between mother and other household members can play a critical role in increasing mothers’ DD. Providing care facilities and counseling to mothers about the nutritional value of consuming different food groups can substantially improve the situation.

## 1 Introduction

Dietary quantity and diversity must be adequate throughout a person's life in order to maintain health and allow for proper physical and mental development. Mothers in developing countries, including Bangladesh, are nutritionally vulnerable due to an undiversified diet, and micronutrient requirements [1,2]. Becoming a mother is a significant life transition for women, which in turn brings substantial life changes in the form of physical changes (biological fact) and new responsibilities (cultural norms). In Bangladesh, there is evidence that mothers have a psychosocial tendency to sacrifice food and resources for the sake of their children and other household members. This affects not only the women's health and survival, but also the health and survival of future generations. In this respect, quantity and diversity of food consumed by mothers is of the utmost significance.

Dietary diversity (DD) and healthy dietary patterns of mothers are necessary for the health and nutrition of the mother and child [3]. DD is defined as the number of food groups consumed across and within food groups during a given time period [4,5]. DD is the most important aspect of diet quality because it provides adequate amounts of micronutrients to meet the nutritional needs of mothers [6]. Low dietary diversity is a major health concern in resource-constrained environments across the world [5]. Previous research suggested that maternal dietary inadequacy contributed to 7% of the global disease burden and at least one-fifth of maternal deaths and poor maternal outcomes [7,8]. Malnutrition is a double burden for mothers because it affects the quality of breast milk, jeopardizing their child's nutritional status, and causing childhood health problems [9,10]. Low-quality and monotonous diets are the norms in developing countries such as Bangladesh, resulting in inadequate nutrition for mothers [11]. According to studies, 59% of women in Bangladesh consume an inadequately diverse diet, and poor DD reflects the overall nutritional status of reproductive-age women [12]. Previous researches have also found that micronutrient deficiencies, such as zinc and iodine, are particularly prevalent in South Asian and Sub-Saharan African countries due to a lack of vegetables, fruits, and animal-source foods in their diets and a reliance on grains and tubers as staple foods [13]. Diversifying mothers' diets by consuming different food groups can help to reduce malnutrition [14]. Therefore, identifying the potential factors associated with mothers' DD can thus help to improve the nutritional and health status of both mother and child.

Numerous studies have examined the factors associated with mother's dietary diversity [5,13,15]. Previous researchers found that socio-demographic characteristics of households, economic factors, food security status, and a lack of nutrition counseling were major factors influencing DD among pregnant women in developing countries [[16], [17], [18], [19], [20], [21]]. Few studies suggested that socioeconomic factors like women's educational status, employment status, monthly income, household assets, family size, information about nutrition, status of sanitation, and household food insecurity were significantly associated with lactating women's dietary diversity [10,22] in Sub Saharan African countries. A study conducted by Shamim et al. [16] found that educational achievement, husbands' occupation, and family size were the major determinants of pregnant women's DD in rural Bangladesh.

According to the preceding discussion, the majority of previous studies dealt with DD in pregnant women. Few studies have been conducted in Sub-Saharan African countries to identify the factors associated with DD in lactating women. There are limited studies on mothers DD in the South Asian context, particularly in the Bangladeshi context. Few studies in Bangladesh have identified the factors influencing rural women's DD. As a result, this study adds to the existing literature by determining the DD of mothers in urban setting and identifying the drivers of DD.

Given this context, the overall goal of this study was to evaluate the DD status of mothers with 6–59 months children in a mega city of Bangladesh. The specific research questions addressed by this study were: (i) what is the DD situation among mothers living in Dhaka city? and (ii) what are the drivers of DD among the mothers? The findings of this study would help design and implement programs to promote a diverse diet for mothers in urban setting in developing countries like Bangladesh and might help in achieving sustainable development goals in an integrated manner.

## 2 Methodology

### 2.1 Study area

**Fig. 1:**
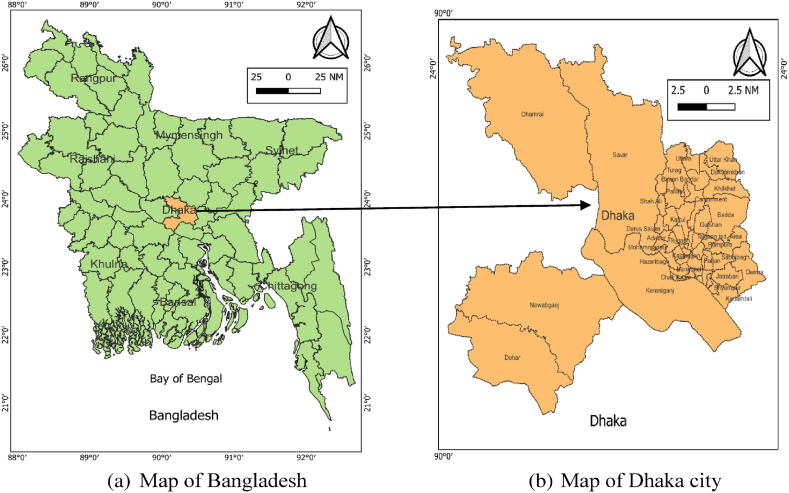
Map of the study area.

This study was conducted in Dhaka city of Bangladesh. Dhaka, the capital of Bangladesh, mentioned as mega city, is characterized by high level of poverty, social vulnerability, shortage of housing, infrastructure and social services (electricity supply, gas and fuel supply, water supply, sewerage and excreta management, and solid waste management), inefficient urban management, and a permanent destination for various rural migrants [23,24]. Therefore, determining the DD and their associated factors in such a mega city could provide policy insights to ensure the DD and nutrition of urban mothers in other developing countries like Bangladesh. Fig. 1 shows the map of the study areas.

### 2.2 Sampling technique and sample size

This study used population information from the Population and Housing Census to select the sample [25]. According to the census, there are 4,681,930 females in Dhaka. About 22.85% of them are under the age of 15 and 13.64% are over the age of 50. According to Bangladesh Health and Demographic Survey data, the majority of women gave birth at the average age of 18, and 90% of women gave birth before the age of 49 [26]. Thus, the study considers women aged 15 to 50 as the target population, which is 63.51% of total female. Using this subset of female as the population (N), the study determined that at least 384 women needed to be surveyed. The following formula shown in Eq. (1) was used to determine the optimum sample size [27]:

(1) $$n=N\times \frac{\frac{Z^{2}\times p\times \left(1-p\right)}{e^{2}}}{N-1\times \frac{Z^{2}\times p\times \left(1-p\right)}{e^{2}}}$$

Where, n = sample size.

N= Population size (2973494).

Z = precession level at 95% confidence interval (1.96).

p = proportion of the population (50%)

e = error margin (5%)

However, the study selected and surveyed 613 mothers at least one biological child aged 6–59 months using a simple random sampling technique. Mothers who were pregnant at the time of survey or who had abnormal/autistic children were excluded from participating in the survey. Households with temporary guests, as well as those celebrating any occasion involving unusual food consumption, such as fasting, Eid, birthdays, or marriage ceremonies, were excluded.

### 2.3 Measurement of dietary diversity

The main outcome variable was mothers' DD, which was measured using the 24-h recall period method. The standard guideline for estimating mothers’ DD had been followed. All of the food consumed by mothers over a 24-h period was divided into the nine categories listed below (Table 1). If a mother consumes a specific food from any group, she receives a score of 1 and otherwise 0. The study excluded double counting of the same food group in order to avoid overestimation of DD. When all of the food groups were added together, the score range was 0–9, and this scale was used as the DD score of mothers [28,29].

**Table 1:** Different food groups for calculating mothers' dietary diversity.

Food Group	Food items
Starchy staples	Corn/maize, rice, wheat, sorghum, millet or any other grains or foods made from these, potatoes, yam, or other foods made from roots
Leafy vegetables	Dark green leafy vegetables like jute leaf, spinach etc.
Vitamin A-Rich fruits and vegetables	pumpkin, carrot, or sweet potato that are orange inside + other Vitamin A rich vegetables (e.g., red sweet pepper)
	ripe mango, ripe papaya, and 100% fruit juice made from these + other locally available vitamin A rich fruits
Other fruits and vegetables	Other vegetables (e.g., tomato, onion, eggplant) + other locally available vegetables
	Other fruits, including wild fruits and 100% fruit juice made from these
Organ meat	Liver, stomach
Meat and fish	Beef, lamb, goat, chicken, duck, other birds, fish, dry fish
Eggs	Eggs
Legume, nuts and seeds	Beans, peas, lentils, nuts, seeds or foods made from these
Milk and milk products	Milk, yogurt or other milk products

### 2.4 Calculation of wealth index

Based on household assets, this study's wealth index represented five social class categories. Table 2 shows the variables that were used to calculate the wealth index for each household. The presence of such assets and a positive response to household utilities resulted in a value of 1, otherwise a value of 0. The asset weights of the first component were then used in principal component analysis and classified into five classes. Several previous studies used five socioeconomic status categories based on the asset weight distribution quintiles [31,32].

**Table 2:** Types of assets used to measure wealth index.

Productive	Non- Productive	Household utilities and other
Sewing machine	Radio	Water supply
Auto rickshaw	Refrigerator/Fridge	Flooring
	TV	Toilet
	Bicycle	Roof materials
	Motorbike	Wall's materials
	Car/Motor Vehicle	Light source/electricity
	Bed	Persons sleeping per room
	Almirah/Showcase/War drop	
	dressing table	
	Dining Table	

### 2.5 Analytical techniques

The study used descriptive statistics and regression analysis to achieve the objectives. The nature of the distribution of dependent variable was evaluated prior to fitting the regression model. The study was unable to categorize mothers' DD into low, medium, or high categories, or to use the FAO [30] minimum dietary requirements cutoff, because no mothers met the criteria for high DD. The poison regression model was also inconclusive and unsuitable for the study. Thus, the study normalized the data by log transformation of DD and conducted log linear regression model using ordinary least square (OLS) approach due to its simplicity and practical applicability [33]. The empirical equation of the model is shown in Eq. (2):

(2) $$logY=\beta_{0}+\beta_{k}X_{k}+\ldots +\mu_{k}\;\left[k=1,2,3,\ldots \right]$$

Where, Y = dependent variable

$\beta_{0}$ = intercept/constant of the model

$\beta_{k}$ = estimated coefficient of exploratory variables

$X_{k}$ = explanatory variables

$\mu_{k}$ = stochastic error terms

As the dependent variable was transformed into log form, the interpretation of the coefficient was [(exp(coefficient)-1) *100] percent changes of dependent variables by a unit change in independent variables. The study tested the multicollinearity among the independent variables. The study fitted five separate models to find the significant relationship between mothers' DD and independent variables because some of the variables, such as mothers' year of schooling, ANC time, improved kitchen, asset access, and wealth index were found to be significantly associated with other independent variables. The variables found to be associated were necessarily important for mothers' DD, as mentioned in previous studies and in light of the current context of the studies. Keeping all other variables unchanged, only these associated variables were kept separately in each model and their effect on mothers’ DD was determined. Finally, to minimize the standard error of the estimated coefficient, a robust model has been fitted and the base model was chosen by imposing criteria of higher R-square and lowest Akaike Information Criteria (AIC). STATA version 17 software was used for all analyses. The description of the independent variables used in the model is given in Table 3.

**Table 3:** Measurement of independent variables.

Variables	Measurement technique	Types
Years of schooling (HHH)	The number of years of formal education received by the household head.	Continuous
Age of mother	Age of the mother in years.	Continuous
Years of schooling (mother)	The number of years of formal education received by the mother.	Continuous
Household size	Number of persons who have resided in the house within the past six months.	Continuous
ANC time	How frequently a mother visited a doctor during her pregnancy for checkups. Even though it might seem difficult to gather accurate information about ANC services received by mothers four or five years ago, the study was able to do so due to mothers' ability for remembering every detail of their pregnancy experience even decades later because it is the most awaited time of their lives. Mothers who did not feel confident answering the survey questions were replaced with another mother who did.	Binary (1 if visited more than 4 times, 0 otherwise)
Improved kitchen	Whether or not a household has a kitchen that has a peeler, blender, mop, rack, etc.	Binary (1 if yes, 0 otherwise)
Access to asset	Whether or not a woman has access to assets such as money, land, jewelry, a bank account, etc.	Binary (1 if yes, 0 otherwise)
Wealth index	A household's social status is determined by a proxy measure of wealth index, which is a categorical variable. Wealth index is divided into five categories such as poorest; lower middle; middle; upper middle and richest.	Categorical
Helping hand	Whether or not a mother have a helping person while cooking, cleaning or other household chores.	Binary (1 if yes, 0 otherwise)
Market access	Whether or not a mother can go to the market when necessary.	Binary (1 if yes, 0 otherwise)
Employment status	Whether or not a mother work outside for daily basis.	Binary (1 if yes, 0 otherwise)

## 3 Results

### 3.1 Descriptive statistics

**Fig. 2:**
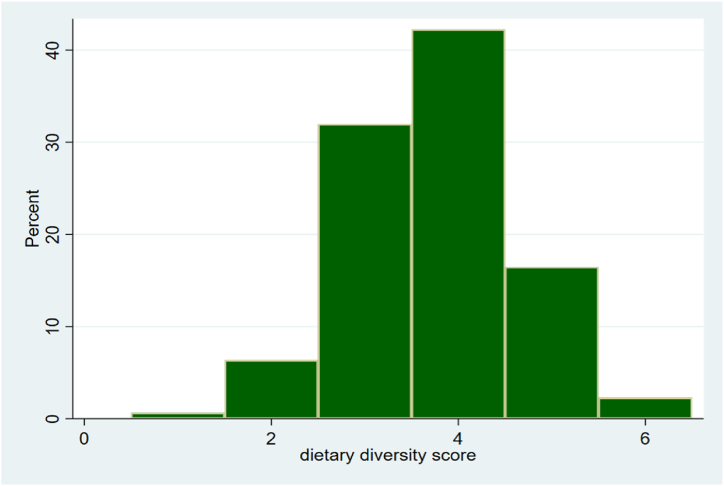
Frequency distribution of mothers' dietary diversity.

Fig. 2 represents the frequency distribution of mothers’ DD. The findings revealed that out of 9 food groups, majority (42%) of the mothers consume 4 types of food groups followed by 3 types of food groups (32%). The results showed general limited DD, with less than 20% of the mothers consuming more than 5 out of the 9 food groups. None of the mothers consumed between 7 and 9 food groups. The maximum DD score was found to be 6 and minimum was 1.

Table 4 represents the characteristics of explanatory variables used in the econometric model. Out of 11 explanatory variables, years of schooling of household head (HHH), age of mother, years of schooling of mother and household size were continuous variables. The findings indicated that the mean year of schooling of the household head was 7.162, which is lower than the mothers’ years of schooling (7.276). The mean age of mothers was found to be 27 years. The average household size was found to be 4.219, with the smallest family having 2 members and the largest having 10 members. Almost 62% of mothers had four or more antenatal care (ANC) visits during their pregnancy. Approximately half of the mothers have an improved kitchen. Although only about one-third of the mothers had access to assets, but in the five quintiles of wealth index, one-third of mothers were from the poorest socioeconomic class, nearly 20% were from the lower middle, middle, and upper middle classes and the remaining 8% were from the richest class. Around 26% of mothers have helping hand to do their household chores and nearly half of the mothers have market access. The proportion of mothers who worked outside the home was 44%, with the remainder being housewives.

**Table 4:** Descriptive statistics of the independent variables used in the model.

Variables	Frequency (percentage)	Mean (SD)	Minimum/maximum
Years of schooling (HHH)		7.16 (5.235)	0/16
Age of mother		27.00 (5.65)	15/45
Years of schooling (mother)		7.27 (4.799)	0/16
Household size		4.21 (1.191)	2/10
ANC time			
<4 times	231 (37.68)		
≥ 4 times	382 (62.32)		
Improved kitchen			
No	302 (49.27)		
Yes	311 (50.73)		
Access to asset			
No	424 (69.17)		
Yes	189 (30.83)		
Wealth index			
Poorest	206 (33.61)		
Lower middle	118 (19.25)		
Middle	119 (19.41)		
Upper middle	119 (19.41)		
Richest	51 (8.32)		
Helping hand			
No	453 (73.9)		
Yes	160 (26.1)		
Market access			
No	310 (50.57)		
Yes	303 (49.43)		
Employment status			
No	342 (55.79)		
Yes	271 (44.21)		

**Note:** Mean (SD) is used for continuous variables; Frequency (percentage) is used for dummy variables.

### 3.2 Factors affecting DD of mothers

After exploring the nature of the studied variables from descriptive analysis, the study moves on to the inferential statistics for digging into the deep. Coefficients and their direction from the different models were almost similar, confirming the robustness of the findings. The significant F-value also indicates good fit of the model (Table 5).

**Table 5:** Determinants of mothers’ dietary diversity.

	Model 1		Model 2		Model 3		Model 4		Model 5	
Variable	Coeff (SE)	Percentage Change^Sig^	Coeff (SE)	Percentage Change^Sig^	Coeff (SE)	Percentage Change^Sig^	Coeff (SE)	Percentage Change^Sig^	Coeff (SE)	Percentage Change^Sig^
Years of schooling (HHH)	0.009 (0.003)	0.90***	0.013 (0.002)	1.31***	0.01 (0.003)	1.01***	0.011 (0.002)	1.10***	0.007 (0.003)	0.70**
Age of mother	0.001 (0.002)	0.10	0.001 (0.002)	0.10	0.001 (0.002)	0.10	0.001 (0.002)	0.10	0 (0.002)	0.00
Years of schooling (mother)	0.007 (0.004)	0.70*								
ANC time (≥4 times)			0.05 (0.024)	5.13**						
Improved kitchen (yes)					0.073 (0.027)	7.57***				
Access to asset (yes)							0.097 (0.027)	10.18***		
Wealth index										
Lower middle									0.012 (0.033)	1.21
Middle									0.127 (0.035)	13.54***
Upper middle									0.136 (0.039)	14.57***
Richest									0.155 (0.048)	16.77***
Household size	−0.001 (0.009)	−0.09	−0.003 (0.009)	−0.29	−0.005 (0.009)	−0.49	−0.003 (0.009)	−0.29	−0.006 (0.009)	−0.59
Helping hand (yes)	0.064 (0.032)	6.60**	0.061 (0.032)	6.29*	0.062 (0.031)	6.39**	0.06 (0.031)	6.18*	0.058 (0.031)	5.97*
Market access (yes)	0.003 (0.021)	0.30	0.009 (0.021)	0.90	−0.003 (0.021)	−0.29	−0.02 (0.022)	−1.98	−0.009 (0.021)	−0.89
Employment status (yes)	−0.059 (0.029)	−5.73**	−0.053 (0.030)	−5.16*	−0.059 (0.029)	−5.72**	−0.057 (0.029)	−5.54**	−0.054 (0.029)	−5.26*
Constant	1.16 (0.059)	218.99***	1.156 (0.059)	217.72***	1.181 (0.085)	225.76***	1.2 (0.059)	232.01***	1.205 (0.059)	233.67***
R-square	0.107		0.109		0.114		0.121		0.137	
F-test	10.358		10.542		11.078		11.9441		9.55	
Prob > F	0.000		0.000		0.000		0.000		0.000	
Akaike crit. (AIC)	70.87		69.71		66.33		60.932		55.985	
Bayesian crit. (BIC)	106.22		105.06		101.68		96.279		104.587	

Note: Coeff. indicates coefficients; SE indicates standard error; ***p < .01, **p < .05, *p < .1.

The findings indicate that on an average if the household head had additional one year of formal schooling, then the mothers' DD would increase by 0.70–1.31% based on different models. Year of schooling of mothers found significantly and positively associated with her own DD. On an average, DD of mothers will increase 0.70% if she received one more year of formal schooling. Receiving ANC for four or more times during pregnancy by mothers has significant positive impact on DD. The DD is 5.13% higher for mothers who receive ANC four or more times than those who receive ANC fewer than four times (model 2). Having an improved kitchen in the household could provide advantages to cooking. The study found that mothers from households having an improved kitchen have higher DD compared to those mothers who didn't have an improved kitchen in their household (model 3). Access to assets is significantly and positively associated with mothers' DD in model 4. It represents that, for the mothers with access to assets, DD is 10.18% higher compared to mothers who didn't have access to assets. Mothers belonging from middle, upper middle and richest class have a higher proportion of DD compared to mothers who belong in lower socio-economic class (model 5). Having a helping hand refers to a person/maid or any eligible members of the household who can assist mothers with their daily household chores. The findings suggested that the presence of a helping hand increased the mother's DD by 5.97% when compared to mothers who did not have a helping hand. Finally, the employment status of mothers had negative association with their DD. Compared to housewife mothers, a mother's DD is reduced by about 5% if she works outside the home (Table 5).

## 4 Discussion

According to the findings of this study, the majority of respondents have lower DD, which is consistent with the findings of previous studies [22,34]. Previous research suggested that lower DD among mothers could be a cause of maternal malnutrition [22]. Mothers require diversified food during the lactation period due to physiological conditions. However, in developing countries such as Bangladesh, the importance of diverse food is frequently overlooked [12]. Greater emphasis should be placed on raising awareness in order to ensure that mothers' diets are diverse and nutritious.

According to our regression analysis, the education of the mother and household head positively influenced the mother's DD. Previous studies [19,35,36] also found an association between education and DD. This could be due to the importance of education in improving knowledge of DD and long-term behavioral change. Educated mothers may make better food choices because they understand the importance of nutrition and are more likely to understand educational messages transmitted through various media channels, which may play a role in increasing DD. Sinharoy et al. [37], indicated that the link between schooling and DD is direct and indirect, through women's voice with husband, where they found that women in Bangladesh with post-primary schooling were more likely to be better able to negotiate improved diets for themselves.

The findings also suggested that when mothers visit a health facility for ANC, they receive various nutritional information and counseling, which may help them improve their DD [36]. According to Tilahun & Kebede [5], mothers who received nutrition information were 2.2 times more likely to meet the minimum DD than those who did not receive nutrition information. Desta et al. [15] found that mothers who had four or more ANC visits had higher DD than those who had one ANC visit.

According to the findings, DD was significantly associated with the wealth status of the household. The likelihood of consuming a variety of foods increases as the wealth index rises Past studies conducted in low- and middle-income countries found that higher socioeconomic status was related to a higher DD score [7,38,39]. This may be because people with lower socioeconomic status tend to eat less of variety of food groups. Previous studies have also found that the socioeconomic status of the household is directly related to the likelihood of a mother having a diverse food [40,41]. Households in better economic standing can afford foods from the markets that are not available at home.

The findings also revealed that mother involvement in service reduces DD. This could be because employment reduces women's available time for activities that improve nutrition and health [42,43]. Komatsu et al. [44] found that working mothers face severe time trade-offs as a result of their heavier burden of unpaid work, and because their paid and unpaid work is frequently undertaken concurrently. The time constraints can be minimized by the presence of other household members as helping hand who can take up works that cannot be performed by a working mother due to increased workload [45]. Our findings also suggested that assisting mother in household work increases DD (Table 5). Studies also found that husbands' engagement in household works significantly increases intake of micronutrient supplements and DD of mothers [46]. Therefore, redistributing household workload with spouse and other household members can help to improve DD.

## 5 Conclusions

Achieving DD is essential for mothers in order to achieve better maternal and child health outcomes. This study identified the factors that contribute to mothers' DD in urban areas in order to design and implement programs to promote a diverse diet for mothers in developing countries such as Bangladesh. The study found that education, access to assets, household wealth status, and ANC services positively influenced mothers' DD, whereas mothers' employment had a negative impact on DD. The findings indicated that there is a need to improve DD, as well as raise awareness about the nutritional benefits of diverse food groups for mothers. It is necessary to improve health-care facilities and educate mothers about the nutritional value of different food groups. Mothers control/access to household assets may amplify nutritional status. An integrated approach to promoting mothers’ nutrition is required.

Although this study provides some useful information, it also has some limitations. This study was limited to a single urban area. Future research may examine additional urban areas in Bangladesh to obtain a comprehensive picture. In the future research, more intensive measure of the influencing factors needs to be addressed while considering the consumption pattern of different groups and places. More complex relationship of mother's DD could be explored by mediator analysis and adopting advanced econometric modeling.

## 6 Ethical consideration

The review board of the Bangladesh Agricultural University Research System, Mymensingh, Bangladesh, granted ethical approval. The approval number is BAURES/ESRC/ECON/20/2020. Each respondent provided verbal informed consent after being informed of the study's objectives, its significance, and the variety of information required. Participation in the survey by respondents was voluntary. Respondents were free to refuse or discontinue the interview at any time. If a respondent refused to be interviewed, another respondent was contacted.

## Funding

This study was supported by Meeting the Under Nutrition Challenge (MUCH) project of the Food and Agriculture Organization of the United Nations (FAO), Bangladesh. Grant number: GCP/BGD/063/EC/1506544.

## Declarations

### Author contribution statement

Sadika Haque: Conceived and designed the experiments; Performed the experiments; Wrote the paper.

Md. Salman; Md. Sadique Rahman: Analyzed and interpreted the data; Contributed reagents, materials, analysis tools or data; Wrote the paper.

Abu Torab M. A. Rahim: Performed the experiments; Analyzed and interpreted the data.

Md. Nazmul Hoque: Performed the experiments; Contributed reagents, materials, analysis tools or data.

### Data availability statement

Data will be made available on request.

## Declaration of competing interest

The authors declare that they have no known competing financial interests or personal relationships that could have appeared to influence the work reported in this paper.
