# Supplementary material for: Mothers’ dietary diversity and associated factors in megacity Dhaka, Bangladesh
Source: Heliyon. 2023 Aug 12;9(8):e19117. doi: 10.1016/j.heliyon.2023.e19117 (PMC10450986; doi:10.1016/j.heliyon.2023.e19117)
Supplement: Multimedia component 1 [file mmc1.pdf]

Interdisciplinary Institute for Food Security (IIFS)

Bangladesh Agricultural University, Mymensingh – 2202

The Interview Schedule on

Mothers' dietary diversity and associated factors in megacity Dhaka, Bangladesh

[N. B. This survey needs to be filled by/for mothers who have children under 5]

Name of interviewer:

Mobile No:

Section A: General Household Characteristics

1. General Particulars:

- **Working mother** **housewife mother** **Self employed**

a) District: Ward No: Mouza/Union: House & Road No.:

b) Name of the Interviewee : Date:

c) Relation of respondent to infant:____ 1=Mother, 2= Sister, 3=Caregiver, 4=Grandmother, 5= Other

d) Family's religion and ethnicity: ______________ *[0=No religion, 1=Muslim, 2=Hindu, 3=Christian, 4=Buddhist, 5=Other]*

2. Information of Household Members ("please write the household head in serial number 1" and name of the respondent in serial number 2 if respondent will no head of household)

SL No.	Name	Age	Relationship with the child	Years of schooling	Occupation (Primary)	Occupation (Secondary)
1.						
2.						
3.						
4.						
5.						
6.						
7.						
8.						
9.						
10.						

*Relationship: ১=বাবা, ২=মা, ৩=ভাই, ৪=বোন, ৫=দাদা/নানা, ৬=দাদী/নানী, ৭= ফুফু/খালা, ৮=চাচা/মামা, ৯= অন্যান্য আত্মীয় স্বজন, ১০=সম্পর্ক নেই, ৯৯=জানিনা

3. Housing:

A. Area (filled in by enumerator): Posh/ middle class/ slum/ any other type (please specify

Tenancy of house: Owned/Rented

Sl.	Question	Answer
**1.**	Total number of rooms:	
**2.**	Does the house have electricity connection? 1= Yes; O= No	
**3.**	What is the construction material of the walls of the main room?________ *0=No walls (less than 2 walls) / Fence, 1=Thatch, grass, sticks, branches, 2=Katcha (bamboo with/out mud, packed mud, betel nut wood), 3=Tin/wood plank, 4=Pakka (cement, brick), 9=Don't know*	
**4.**	What is the construction material of the roof of the main room? *0=No roof/plastic, 1=Thatch or grass, 2=Tin, 3=Pakka (cement/tile), 9=Don't know*	
**5.**	What kind of toilet facility do members of your household usually use? (*1-Piped sewer system, 2- Septic tank, 3 -Off-set pit latrine with slab, 4 – Direct pit latrine with slab, 5 – Direct pit without slab, 6. Dual pit latrine, 7. Hanging toilet, 8. Other……,*	

4. Household Assets :

Sl.	Name of the Assets	Number	Sl.	Name of the Assets	Number
1.	Almirah/Showcase/ waredrobe		11.	Clock (Wall/Table/ wrist watch	
2.	Dressing Table		12.	Autorickshaw	
3.	Bed		13.	Sewing machine	
4.	Sofa		14.	Motorcycle	
5.	Dining Table		15.	Vehicle/ Car	
6.	Reading Table		16.	Mobile phone/ land phone	
7.	Computer/ laptop		17.	Generator/ IPS	
8.	Refrigerator/ Fridge		18.	Washing Machine	
9.	Fan		19.	TV	
10.	Bicycle				

Section B. General Cleanliness

1. Drinking water

No.	Questions	Code/ Answer
1.	Main source of drinking water: (1= Supply water, 2= Deep Tube well (>250 feet), 3= Shallow Tube well (<250 feet), 4= Pond/river, 5= Waterfall/spring, 6= Rainwater harvesting, 7=Other, specify)	
2.	Is the water source tested for arsenic? *(1=yes, 0 = No, 2=don't know)*	
3.	Do you normally treat drinking water? (1=yes, 0 = No)	
6.	How often do you clean the water storage container? *[1= everyday, 2= every alternate day, 3= twice in a week, 4= once in a week, 5= Don't know*	

2. Washing Hands

No	Questions	Code/ Answer
1.	*What do we need to wash our hands?(1= water and soap, 2 = water*)	
2.	Do you think washing hand with soap is always necessary? (*1=Yes 0=No*)	
4.	Is water available inside/ just outside the toilet for washing hands after defecation? 1 = inside, 2 = outside	
5.	Do you know what would happen if we do not wash our hands? 1=we can get sick, 2=bacteria and germs can be transferred inside of our mouth, 9=don't know	

3. Sanitation and hygiene

No	Questions	Code/ Answer
1.	Where does the child defecate? in toilet/potty/in the dwelling/in the diaper/in the cloths	
4.	How often do you dispose solid waste? [1= daily, 0= not daily]	
5.	Do you sometimes see flies in the area where the food is kept/ stored? 1=yes 2=No	
6.	How often do you trim fingernails? [1= once in a week, 0= if more than a week]	

Section C: Ante-natal Care and Maternal Health

Sl No	Question	Code/Answer
1.	At what age did you get married? *(Code: record the completed years)* (If multiple marriages, take the 1st marriage)	
2.	At what age, did you become mother for the first time? *(Code: record the completed years)*	
3.	How many times did you receive antenatal care?<br>*Code: 1-4= number of ANC visit, 5= 5 times or more, 9=Don't know*	

Section D: Measuring Women Empowerment

No	Questions	Answer
1.	Do you own land/house/car/gold/ own bank account (any other thing whose value is more than 50,000 Tk) in your name?	
**2.**	Can you spend your own money and husband's earned money without seeking husband's permission?	
**3.**	Do you have access to low cost kitchen equipment which can make your HH chores easier like easy cooking stove like gas (natural or LPG), peeler, blender, pressure cooker, fry pan, mops, etc?	
4.	Does your husband help you in household works?	
5.	When he is angry for any reason, does he quarrel with you/ shout with you/ beat you?	
6.	Who decides what food items to buy from the market?	
7.	Who makes most decisions about what health expenditures to make?	
8.	Can you go to the market when it is needed?	
9.	Can you go to hospital/ clinic without asking anybody?	
10.	Can you participate in community events of any social, political, or religious organizations?	
11.	Who usually helps when you need assistance with the HH chores?	

7. Workload of a mother: On a typical day, how many hours do you spend on the following activities:

SL	Activities	Time
1.	Care of children	
2.	Care of elderly/disabled people or other household member	
3.	Preparing breakfast	
4.	Cooking lunch	
5.	Cooking dinner	
6.	Preparing snacks	
7.	Cleaning the house	
8.	Washing clothes	
9.	Cultivating land	
10.	Tending farm animals	
11.	Formal labour/ office hour	
12.	Other business activities	
13.	Leisure time (e.g. socializing, watching tv)	
14.	Sleeping at night	

SL	Activities	Time
15.	Personal care and rest	
16.	Religious activities	
17.	Tutor the children to do their homework	

Section G: Knowledge and Practice of mother
[Answer for each question on the following] 1=Yes, 0=No

SL	Question(প্রশ্ন)	Answer
1.	Do you try to take at least five colors of food (fruits, vegetables or salad) every day?	
2.	Do you wash vegetables before cutting?	
3.	Do you rinse the water after boiling vegetables while cooking?	
4.	Do all members of your family consume at least one egg every day?	
5.	Do you frequently eat fried foods from restaurants?	
6.	Do you add sugar to the milk of the child?	
7.	Do you change the food menu frequently?	
8.	What should we provide who suffers from diarrhea? (we need to follow if she mentions ORS an fluids)	

Section H: Dietary Diversity

Please describe the foods (meals and snacks) (name and quantity) that you consumed yesterday during the day and night, whether at home or outside the home.

(*Write down all foods and drinks mentioned. When composite dishes are mentioned, ask for the list of ingredients. When the respondent has finished, probe for meals and snacks not mentioned.)*

Breakfast	Snack	Launch	Snack	Dinner	Snack

3. Foods taken in the last two weeks at household (How many times?)

Food items	Times		Food items	Times
Beef/mutton/lamb			Guava	
Chicken/Duck meat			Banana	
Any liver			Lime	
Any kind of fish			Green papaya	
Dried fish			Jute leaf	
Pulses			Cucumber	
Egg			Bitter gourd	
Milk			Okra	
Curd/yogurt			Spinach	
Yam/arum (kachu)			Red amaranth/ Squash	
Sweet potato			Sweet gourd	
Gram/ pea			Palwal	
Potato			Snake gourd/ Ribbed gourd/ Sponse gourd	
Mango			Brinjal	
Jackfruit			Teasle gourd	
Yard long bean				

Thank you very much for your kind attention
